# Supplementary figures and images for: Positional SHAP (PoSHAP) for Interpretation of machine learning models trained from biological sequences
Source: PLoS Comput Biol. 2022 Jan 28;18(1):e1009736. doi: 10.1371/journal.pcbi.1009736 (PMC8797255; doi:10.1371/journal.pcbi.1009736)

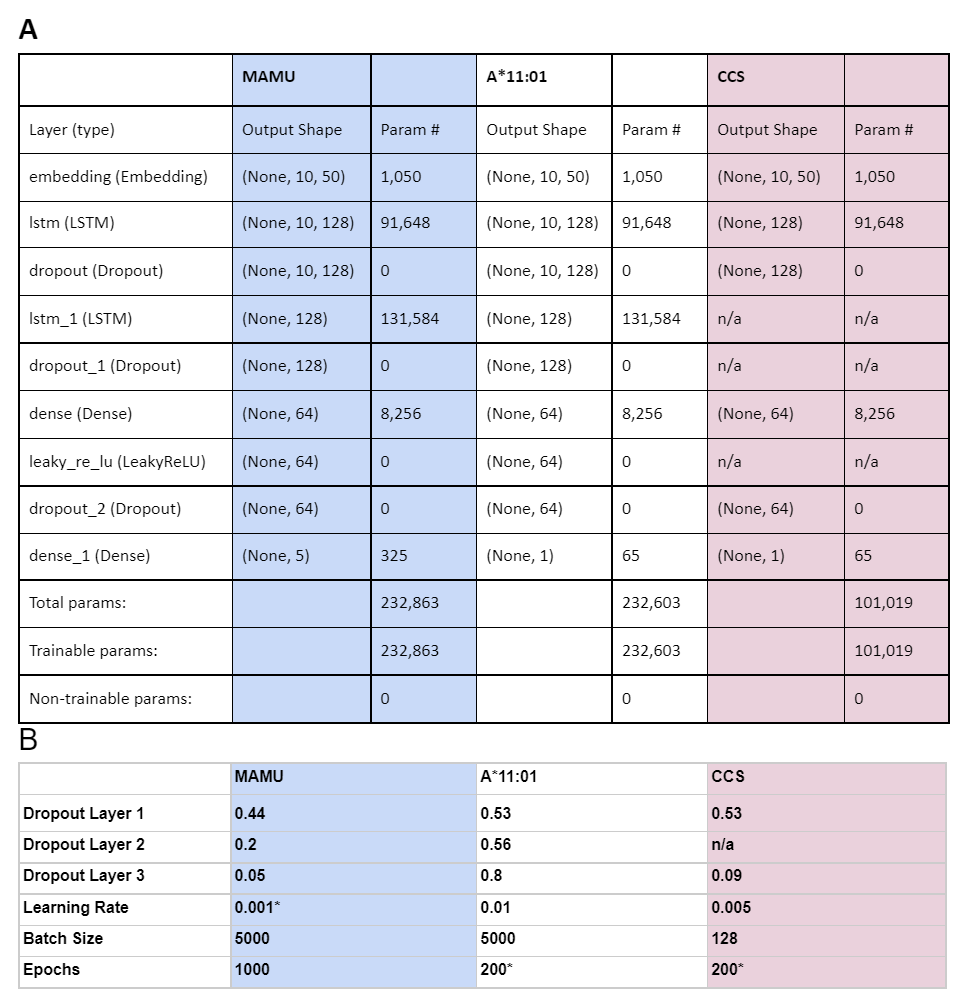

Supplement: S3 Fig — (A) The architecture of the model consists of an embedding layer with 10 inputs with 21 dimensions each, representing each position of the peptide and each of the numeric representations of the possible amino acids and the end marker. This is followed by a pair of LSTM and dropout layers, with the dropout ratios determined by a hyperparameter search. Following the LSTM layers are a dense layer, a leaky ReLU activation layer, a final dropout layer, and a final dense layer with five outputs, each representing the intensity of the corresponding allele. The model was trained with a batch size of 5000 for 1000 epochs. (B) Hyperopt was used to determine the ideal hyperparameters for the model using a tree of parzenestimators algorithm over 100 evaluations. * indicates a hardcoded hyperparameter. (TIF) [file pcbi.1009736.s003.tif]
