# Supplementary material for: Positional SHAP (PoSHAP) for Interpretation of machine learning models trained from biological sequences
Source: PLoS Comput Biol. 2022 Jan 28;18(1):e1009736. doi: 10.1371/journal.pcbi.1009736 (PMC8797255; doi:10.1371/journal.pcbi.1009736)
Supplement: Attachment — Submitted filename: 118147_1_rebuttal_2177190_qzhkb8.docx [file pcbi.1009736.s019.docx]

**Manuscript number:** RC-2021-00899

**Corresponding author(s):** Jesse Meyer

## General Statements [optional]

We thank the reviewers for their critical review of our work. Through addressing these comments, we made at least two very interesting additions to our manuscript. We hope that our responses are satisfactory.

## Point-by-point description of the revisions

Please find our responses to comments on the following pages.

Reviewer #1 (Evidence, reproducibility and clarity (Required)):
In this paper, the authors use a previously published method SHAP for interpreting deep learning (DL) models (specifically LSTMs) that are trained for predicting physicochemical attributes of peptides (such as antigenicity and collisional cross section). The paper shows that it's capable of identifying some amino acid residues contributing to the prediction results of the DL models.

Reviewer #1 (Significance (Required)):
1. One main ideas of the paper is to use SHAP for determine the significant amino acids at each position (or pairs of AA at each position) contributing to the prediction. Some of the interpretation results are consistent with findings reported previously. This is very nice; however, most of these findings are statistical results such "XX is often present at the second position for the peptides with the positive outcome", which are relatively straightforward and may be derived by using some statistical methods without using DL models. We expect more complex patterns can be discovered in addition to these statistical observations.

We thank the reviewers for these comments.

First, to the point about discovering complex patterns, we note that one use of PoSHAP we discuss later in the paper is that PoSHAP enables interposition dependence analysis, which depends on interactions between residues and would not be reflected by summary statistics.

Second, we agree it is important to show whether PoSHAP produces different residue importance maps than simple statistical summaries of amino acids in each group. The strongest binding peptides, or the highest mobility for the CCS model, were determined by taking only peptides that fall above a linear regression best fit of the ranked experimental values. Statistical summary heatmaps were created and then compared to those from PoSHAP revealing some similarities but also many differences. We added the following text and figure to the results section to illustrate these points:

*“We wondered whether the patterns revealed by PoSHAP simply reflect the summary statistics for the high-binding or high-CCS subset of peptides. As expected, due to known differences in amino acid abundance across the proteome, the prevalence of amino acids was different across the training data and were also heterogeneous across positions (Figure 5A). To determine the subset of high CCS peptides, peptides were ordered in the training set by their CCS rank and then linear regression was performed to get the average trend line (Figure 5B). Any peptide above that trendline was defined as “high CCS”, and the frequency of amino acids at each position in this set was summarized using a heatmap (Figure 5C). Compared to the statistical amino acid frequencies, PoSHAP suggests a greater importance to arginine at both termini, the importance of tryptophan to increase CCS becomes apparent, and interior glutamic acid contributes less to high CCS than the frequencies would suggest (Figure 5D). The same analysis was repeated for MHC data (Supplementary Figures 9 and 10). This demonstrates that PoSHAP found non-linear relationships between the inputs and the outputs that are not present by simple correlation. “*

**Figure 5: Amino acid summary statistics differ from PoSHAP values for the CCS data.** (A) Amino acid counts as a function of position for training data. (B) Procedure for picking the ‘top peptides’ with the highest CCS. Linear regression was performed on the peptides ranked by their actual CCS value. Any peptide that fell above the trendline and overall mean were defined as ‘top peptides’. (C) Counts of amino acids for the top peptides were summarized in a heatmap. (D) Mean SHAP values across amino acids and positions from PoSHAP analysis.

We also added the corresponding supplemental figures showing the same examples for the MAMU A001 model and human MHC models:

**Supplemental Figure 9: Amino acid summary statistics differ from PoSHAP values for the A001 MAMU MHC I data.** (A) Amino acid counts as a function of position for training data. (B) Procedure for picking the ‘top peptides’ with the highest CCS. Linear regression was performed on the peptides ranked by their actual CCS value. Any peptide that fell above the trendline and overall mean were defined as ‘top peptides’. (C) Counts of amino acids for the top peptides were summarized in a heatmap. (D) Mean SHAP values across amino acids and positions from PoSHAP analysis. For the MAMU model, the amino acid frequencies of the input peptides show no obvious preference for amino acid position, but some amino acids are over-represented overall. The presence of the “end” token is more likely to be a high binder statistically (C), but the PoSHAP reveals that this end token is not the main determinant of binding (D).

**Supplemental Figure 10: Amino acid summary statistics differ from PoSHAP values for the human A1101 MHC I data.** (A) Amino acid counts as a function of position for training data. The distribution of amino acids in this data. (B) Procedure for picking the ‘top peptides’ with the highest CCS. Linear regression was performed on the peptides ranked by their actual CCS value. Any peptide that fell above the trendline and overall mean were defined as ‘top peptides’. (C) Counts of amino acids for the top peptides were summarized in a heatmap. (D) Mean SHAP values across amino acids and positions from PoSHAP analysis. There are clear differences between the summary statistics of top peptides (C) and PoSHAP heatmap (D). For example, the end token is prominent in the summary statistics absent from the PoSHAP interpretation. Also, the preference for S/T/V at position two is tempered according to PoSHAP, but would be determined to be very important by the summary statistics.

2. Although the interpreting results reported in the paper largely agree with previous reports, the paper did not explicitly model the frequency of different amino acid in the training data. For instance, if the amino acid 'A' happens to be over-represented in the positive samples of peptides in the training data, the DL model may consider it as to contribute to the positive prediction, which may not be not true. This issue might become more serious when pairs of amino acids are considered. The authors may want to analyze this potential issue in their results.

We agree and understand the concern for the overrepresentation of amino acids that might skew the training of our models. To determine if this is an issue, as part of the response to the previous question, we looked at the amino acid counts for all peptides (Figure 5A, Supplemental Figures 9A and 10A). In general, the PoSHAP heatmaps (panel Ds in the same figures) look very different from the frequencies of amino acids (panel Cs in the figures).

3. Even on a balanced training dataset, the LSTM model to be interpreted may still contain arbitrary bias due to invertible overfitting, which the authors did not discuss. It will be more convincing by training multiple models using different hyper-parameters and optimization algorithms, and then see if similar interpretation results can be reached among most or all of these models.

We assume the reviewer meant ‘inevitable overfitting’ instead of “invertible overfitting”? If so, the original manuscript did assess overfitting in Figure S4 based on the training and validation loss over training epochs.

We think the reviewer makes a good point that different models might produce different interpretations, so we trained new models without optimization and with different hyperparameters and with a different optimizer (RMS prop). We see essentially the same PoSHAP interpretations. We added the following text to the results section along with these three new supplemental figures:

*“Given the dependence of the model interpretation results on the model used, the same model architecture trained with different parameters might result in different model interpretation. Given this, models for each of the three tasks mentioned here were retrained with different hyperparameters including the “RMS prop” optimizer. Each model produces similar or better prediction performance compared to the earlier version, and the model interpretation by PoSHAP was almost identical to the previous results in all three cases (Supplementary figures 12, 13, 14). This suggests that the model architecture drives the differences in interpretation, not the model training process.”*

**Supplemental Figure 12. PoSHAP Analysis of Mamu A001 With Unoptimized Hyperparameters and RMSprop.** A new model for the Mamu data was trained using the same architectures but with different hyperparameters and RMSprop as the optimization algorithm. Loss was plotted as mean squared error compared to the validation data. (A) Similar metrics for MSE, r, and p-values were obtained (B). Similar patterns are also observed for the PoSHAP heatmap of A001. (C) A dependence plot for A001 shows similar patterns to the Adam optimized model, including the positional dependence of proline at position two for high SHAP values of serine and threonine.

**Supplemental Figure 13. PoSHAP Analysis of A:11*01 With Unoptimized Hyperparameters and RMSprop.** A new model for the A:11*01 data was trained using the same architectures but with different hyperparameters and RMSprop as the optimization algorithm. Loss was plotted as mean squared error compared to the validation data. (A) Similar metrics for MSE, r, and p-values were obtained (B). Similar patterns are also observed for the PoSHAP heatmap of A:11*01. (C) The SHAP ranges by position plot for A:11*01 shows similar patterns to the Adam optimized model, including the largest range of SHAP values at position two, nine, and ten.

**Supplemental Figure 14. PoSHAP Analysis of CCS With Unoptimized Hyperparameters and RMSprop.** A new model for the CCS data was trained using the same architectures but with different hyperparameters and RMSprop as the optimization algorithm. Loss was plotted as mean squared error compared to the validation data. (A) Similar metrics for MSE, r, and p-values were obtained (B). Similar patterns are also observed for the PoSHAP heatmap of CCS. (C) Dependence analysis was performed on the dataset and the combined distance-interaction type bar plot shows similar relationships between the groupings, notably charge repulsion’s split.

4. For the dependence analysis, it is not completely clear why the distance is used as the variable, while the relative position of the amino acid residue in the peptide is ignored. For example, if there is a strong interaction between the first and the last residues in the peptide, their distance changes depending on the peptide length. In figure 6, the authors showed strong interactions between amino acid that are 8-9 residues apart may suggest the peptide length actually plays a role here.

We used distance because as the dependence analysis is a calculation of the difference in means between two distributions of SHAP values, dependent of the amino acid at another position. We believe that the distance between these interacting points is a natural choice and among the most informative metrics to explain these interactions. We agree with the reviewer that peptide length is important to the magnitude of the interactions between amino acids. We also recognize that there may be interactions between the peptide termini that could be obscured by the interactions of the longer peptides. To better explore this possibility, we performed the dependence analysis on each of the different peptide lengths separately, to see if this is the case. Unfortunately, given the smaller size of these data subsets, we were unable to show significant differences in the interaction groupings. Though, interestingly enough, the significant interactions for the peptides of length eight only occurred between neighboring amino acids or the termini. This may suggest an interaction that could be explored in the future.

We added the following text and supplemental figure 11 to the results:

*“Finally, to try to ask if the absolute positions of amino acids in the peptide are relevant for the interaction, the data was split into 8, 9, or 10mers before analysis (Supplemental Figure 11). This revealed that there may be interactions between the termini, but this effect may be difficult to observe because there are significantly fewer 8mers and 9mers in the CCS dataset.”*

**Supplemental Figure 11.** **SHAP Values of Collisional Cross Section by Peptide Length.** The impact of peptide length on SHAP values was explored for the CCS data. The dataset was split into peptides of length 8, 9, and 10. All SHAP values were plotted as violin plots. The mean SHAP values were plotted in heatmaps by position and amino acid and standardized. Significant interactions by dependence analysis were plotted in bar charts by distance between interactions.

To further support our decision to use distance as an interaction metric, we have also now included an additional box plot for Figure 7, demonstrating the interactions between each of the categories combined with distance. We have found that some of the bimodality of the interaction categories are explained by the distance at which they interact. Most strikingly is charge repulsion that decreases CCS when neighboring but increases CCS when the interaction is further.

We added the following text and updated Figure 7 to the results section:

*“Additionally, there are interesting differences in the interactions of the amino acid among the significant set of interactions* ***(Figure 76B).*** *All significant interactions from the CCS data (****Supplemental Table 3****, adj. p-value < 0.05) within the peptides were grouped by the expected interaction type occurring between the amino acids based off of the chemistry of the R groups. Interestingly, this analysis revealed that generally attractive molecular interactions, including charge attraction and polar interactions on average decreased predicted CCS while generally repulsive molecular interactions, including charge repulsion and “other” interactions (likely steric interactions or interactions between the termini) increased predicted CCS. Notably, there were no significant hydrophobic interactions. This may reflect that hydrophobic interactions between amino acids in a peptide act to minimize contact with a polar solvent, rather than acting as an attractive force itself. As the peptides were measured with solvent, hydrophobic interactions were not present.*

*Though it is evident that the mean of each interaction type corresponds to the expected impact those interactions would have on CCS, each of the interaction dependence plots are bimodal, with some interactions increasing CCS and some decreasing it. To dissect this observation further, we combined the two methods of splitting the data to see if the bimodality of interaction types would be resolved by distance (****Figure 7C****). Though definitive conclusions cannot be made for most categories, likely due to the ever decreasing sample size by splitting, of note is the difference between neighboring charge repulsion and non-neighboring charge repulsion. Neighboring charge repulsion seems to decrease CCS while distant charge repulsion increases CCS (see adjusted p-value from Tukey’s posthoc test in* ***Figure 7D****). When distant, charge repulsion makes intuitive sense as the amino acids are forced apart, linearizing the peptide and increasing the surface area. When neighboring, it is possible that the repulsion causes a kink in the linear peptide, decreasing the cross section. Overall, these analyses demonstrate that the models were able to learn fundamental chemical properties of the amino acids and through PoSHAP analysis we were able to uncover them.”*

**Figure 7. Dependence analysis of CCS model. (A)** Significant (Bonferroni corr. P-value < 0.05) values were taken from the interpositional dependence analysis and the difference in the mean between the interdependent amino acids SHAP values and the remaining amino acids at each compared position pair were grouped based on the distance between the dependent interaction, **(B)** the category of interaction, or **(C)** distance and interaction category. Categories are labelled by the following for the combined bar plot and heatmap: ζ<< = charge attraction, ζ<> = charge repulsion, * = other, and δ = polar. For the distance analysis, interactions were grouped into three categories, neighboring (distance = 1), near (distance = 2, 3, 4, 5,6), and far (distance = 7, 8, 9). * indicates significance (ANOVA with Tukey’s post hoc test p-value < 0.05). For the interaction categories in **(A)** and **(B)**, each interaction was grouped by the expected type of interaction between the two amino acids. Significant differences between interaction types are noted by the pairing by lines (ANOVA with Tukey’s post hoc test p-value < 0.05). **(D)** Significant differences between combined categories are illustrated by the heatmap where significant values (ANOVA with Tukey’s post hoc test p-value < 0.05) are designated by colors other than purple. Exact p-values for each are provided in **Supplemental Table 4.**

5. Also, it would be better to show that how the result looks like when applying this method to peptides in the negative samples (e.g., the peptides that are not bound by MHC in the antigenicity prediction experiment). Will the interpreting results also be negative?

We agree this is an interesting idea. We updated the supplemental figure showing PoSHAP of top peptide subsets to also show PoSHAP of bottom peptide subsets (supplemental figure 8). The results suggest that certain amino acid positions are detrimental to binding, for example D/E at various positions. We updated this section to add:

*“We also performed the same analysis with the eight peptides with the lowest binding predictions (****Supplemental Figure 8)****. These PoSHAP heatmaps are primarily composed of negative SHAP values, suggesting that using this subset reveals amino acids at certain positions that are detrimental to MHC binding.”*

**Supplemental Figure 8. Pooled PoSHAP for bottom and top predicted subsets of the data.** The mean SHAP values for each amino acid at each position were calculated for the peptides with the bottom (A) or top (B) 0.013% predicted intensity (top 8 peptides) for the “A” Mamu alleles. Due to the small sample size, most of the amino acid positions have a value of zero. The positions with extreme values, however, illustrate important amino acids for prediction. Notably for A001 and A002, aspartic acid and glutamic acid contribute to low prediction along the peptide, suggesting charge may inhibit binding. For the top predictions, phenylalanine or leucine are important at the first position for both A001 and A008. A serine or threonine at position two is important for A001, A002, and A008. All alleles demonstrate the importance of a proline near the middle of the peptide.

6. Finally, it will be interesting to see the interpreting results when the method is applied to the DL models on more challenging tasks such as the prediction of tandem mass spectra of peptides. The authors may want to discuss these applications.

We agree it would be very interesting to apply this method to interpret predictions of tandem mass spectra. In this paper we already demonstrated PoSHAP on three different datasets with three different models, so we feel that adding a fourth model is out of the scope of this work. We do agree that we would like to explore this option in the future. We added this idea to the discussion section:

*“Altogether the advances described herein are likely to find widespread use for interpreting models trained from biological sequences, including models not covered here such as those to predict tandem mass spectra (reviewed in 33).”*

I am primarily interested in algorithmic and statistical problems in genomics and proteomics. We have develop deep learning models for predicting the full tandem mass spectrum of peptides, and am interested model interpretation methods to explain the fragmentation mechanism resulting in non-conventional fragment ions in tandem mass spectra of peptides. I review the paper in collaboration with my Ph.D students, who are developing deep learning models for computational mass spectrometry.

Reviewer #2 (Evidence, reproducibility and clarity (Required)):

**Comments to the Authors**

In this study, the authors developed a framework named PoSHAP for the interpretation of neural networks trained on biological sequences. The current manuscript can be stronger if the following issues can be clearly addressed.

1. As interpreting model with SHAP is a vital part of this manuscript, it would be better to provide descriptions of the underlying principles of SHAP to enable the readers to understand the paper easily.

We recognize that understanding the principles of SHAP is vital. To better explain SHAP, we have added the following text to the introduction:

*“SHAP is a perturbation-based explanation method where the contribution of an input is calculated by hiding that input and determining the effect on the output. SHAP expands this using the game theoretic approach of Shapely values that ensures the contributions of the inputs plus a calculated baseline sum to the predicted output.”*

2. It is emphasized in the manuscript that PoSHAP is introduced to interpret neural networks trained on biological sequences. However, it is not clear why the authors choose the Model Agnostic Kernel SHAP, which is based on Linear LIME. Although it can be used for any model, the performance of which may not be optimal. In this regards, perhaps Deep SHAP or Gradient SHAP is more appropriate, both of which are designed for deep learning networks [1]. It would be better to provide some additional experiments on Deep SHAP and this work will be more convincing if the same or similar contribution of each position on each peptide as that of Kernel SHAP.
[1] Lundberg, S., and S. I. Lee. "A Unified Approach to Interpreting Model Predictions." Nips 2017.

Our goal in using KernelExplainer was to demonstrate that PoSHAP was not dependent on model specific interpretation methods. However, we have realized that this intention may not have been clearly stated or demonstrated. To expand on this, we have included a new Figure 8, which shows PoSHAP analysis comparisons to other classes of machine learning models, all using Kernel Explainer. This result was interesting because it revealed that even though the XGboost model technically performed better at prediction (Figure 8A, reduced MSE and higher spearman rho), and produced a similar PoSHAP motif heatmap, the interpositional dependences from the perspective of distance (Figure 8C) or chemical interactions (Figure 8D) were substantially muted. This is also apparent with the other standard machine learning model ExtraTrees. This result shows that the choice of model architecture is important, and this direct comparison would not be possible if we used the DeepExplainer.

We added the following text and figure to the manuscript:

*“ PoSHAP uses the SHAP KernelExplainer method, which is based on Local interpretable model-agnostic explanations (LIME). Using the general KernelExpplainer method enables direct comparison of interpretations produced by different models trained from the same data. To ask whether PoSHAP interpretation changes based on the model used, the CCS data was used to train XGboost or ExtraTrees models. Surprisingly, the XGboost model performed better than the LSTM model with regard to MSE and spearman rho between true and predicted values in the test set (Figure 8A). ExtraTrees was slightly worse than the other two models. The model interpretation heatmaps from PoSHAP were similar between the LSTM and XGboost, but the interpretation from the ExtraTrees model was missing the high average SHAP due to n-terminal histidine or arginine (Figure 8B). Even though XGboost produced a similar PoSHAP heatmap, the interpositional dependence with regard to distance (Figure 8C) and chemical interactions (Figure 8D) was muted. This shows that the choice of model is important for revealing amino acid interactions.”*

**Figure 8. CCS PoSHAP of Various Machine Learning Models.** PoSHAP analysis was performed on two additional machine learning models, Extra Trees and Extreme Gradient Boosting (XGB). Predictions were plotted against experimental values and the Mean Squared Error and r values are reported for each model (A). PoSHAP heatmaps were created for each model (B), illustrating an increase in model complexity as more sophisticated models are used. Dependence analysis was performed on each model and the significant interactions are plotted by distance (C) and by combined distance and interaction type (D).

3. As described in the manuscript, "Correlations between true and predicted values were assessed by MSE, Spearman's rank correlation coefficient, and the correlation p-value." As an important indicator for evaluation, the exact p-values should be provided in the seven subgraphs in Figure 2, not p=0.0.

We agree with the reviewer that reporting accurate p-values can assist in evaluation. We have updated the figures to reflect the p-values as far as we were able to determine them. Unfortunately, we are limited by the nature of the double data type in python and so reported that the p-value was less than the minimum value allowed by a double in six of the seven graphs. Additionally, the scales have been marked symmetrically as you mentioned in comment 4.

4. It should be noted that the coordinate scales of Figure 2B and Figure 2C need to be marked symmetrically. And from Figure 2B, we can see that, the IC50 with smaller (<0) and larger (>0.8) values cannot be well predicted. Can the authors provide a detailed explanation about these results?

We understand the reviewer’s concern with poor prediction of extreme values. Figure B represents the IC50 prediction for the A1101 human allele which was the smallest of the datasets we used for training. It only consists of 4,522 entries, around 1/10 of the data used for the Mamu alleles and CCS. Because of this, it is likely that there were not enough examples of datapoints at the extremes to reliably train the model to account for them. However, given the limited size of the dataset, we were surprised with the satisfactory predictions. More importantly, the purpose of our paper is model interpretation not model prediction accuracy, and this shows that even when predictions are not perfect, the model interpretation by PoSHAP can still be effective. We thank the reviewer for noticing this and added the following statement to the results:

*“Remarkably, this was achieved for A*11:01 using a total dataset of only 4,522 examples, which shows that PoSHAP can be effective with even less than 10,000 training examples. “*

5. References are needed in some descriptions in the manuscript. For example, "one might train a network to take an input of peptide sequence and predict chromatographic retention time", "RNNs have found extensive application to natural language processing, and by extension as a similar type of data, predictions from biological sequences such as peptides or nucleic acids".

We apologize for missing these references. We have now cited these statements and have added many additional references as part of our revision.

6. The description of the adopted three models in the section "Model architecture" is a bit confusing. As described in this section, "The LSTM layer outputs a 50x128 dimensional matrix to a dropout layer where a proportion of values are randomly set to 0", "a second LSTM layer outputs a tensor with length 128 and a second dropout layer then randomly sets a proportion of values to 0". But as shown in the Supplemental Figure 3, the output size of the first LSTM was 10x128. Also, as shown in Table 1, the dropout rates were not 0. Therefore, the section should be adjusted for clear clarification.

We apologize for the confusing wording. We meant that dropout layers randomly set values=0, not that the dropout proportion was 0. We reworded this part to read:

*“The LSTM layer outputs a 10x128 dimensional matrix to a dropout layer where a proportion of values are randomly “dropped”, or set to 0. For the MHC models, a second LSTM layer outputs a tensor with length 128 to a second dropout layer. Then in all models, a dense layer reduces the data dimensionality to 64. For the MHC models, the data is then passed through a leaky rectified linear unit (LeakyReLU) activation before a final dropout layer, present in all models.”*

Reviewer #2 (Significance (Required)):

Pls refer to my comments provided as above.

Reviewer #3 (Evidence, reproducibility and clarity (Required)):

**Summary:**

The main goal of the work is to provide the interpretation of Deep Neural Networks (LSTM in the paper) trained on biological sequences. For this purpose authors used the framework introduced earlier - SHapley Additive exPlanations (SHAP), in particular - the slight adaptation of this method called positional SHAP (PoSHAP), because they are interested in the impact of each position of the input sequence to the model output. They demonstrate this on three regression tasks that predict peptide properties.

**Major comments**

The main contribution, highlighted in the paper: authors showed how PoSHAP discloses amino acid motifs that influence MHC I binding. Further they described how PoSHAP enables understanding of interpositional dependence of amino acids that result in high affinity predictions. Also they argued that this work also contributes to a method for accurate prediction of peptide-MHC I affinity using peptide array data enabled by novel application of a neural network that combines amino acid embedding and LSTM layers.

There are some comments about the statements above:

1.Why was the LSTM model chosen? Recent publications showed the success of the Transformer model for biological sequences; however this direction was not covered in the related work overview. The architecture choice then should be better justified. Also the choice of LSTM for the biological sequences is not new and authors should better claim their statement about "novel application of a neural network that combines amino acid embedding and LSTM layers ". Where exactly is the novelty? Could the community use the pretrained embeddings for their purpose?

The reviewer is correct that transformer models are highly effective for making predictions from biological sequences. In fact, many models do well, and there is no single correct choice of model for this task. Though there are many models to choose from, our models are sufficiently accurate. Importantly, the main contribution of our manuscript is not to train the most accurate models, but rather to demonstrate a strategy for positional model interpretation based on SHAP. Related to that point, please note our response to reviewer #2’s second comment that our approach uses the kernel explainer and can be applied to any model. However, we do agree that we neglected coverage of the transformer model in the introduction and have added a paragraph to the introduction covering some of the recent work in this area:

*“Many effective deep learning model architectures are available for making predictions from inputs of biological sequences, and there is currently no single correct choice. CNN models such as MHCflurry 2.0 (40) and LSTM models are effective at predicting MHC binding of peptides (41). Even simpler models, such as random forests, have been used to predict MHC binding (42,43). Prediction of other peptide properties like tandem mass spectra are often done with CNN or LSTM models (33). More recently, given the extraordinary performance of transformer models like BERT (44) and GPT-3 (45) for NLP, there is interest in transformer models for biological sequences (46).”*

We also want to be sure we do not overstate the novelty of our contributions. We have updated our discussion to better reflect the nature of our contributions. We reworded the statement quoted above to read:

*“Overall, the three modeling examples laid out herein serve as a tutorial for PoSHAP interpretation of almost any model trained from almost any biological sequence.”*

2. The attention mechanism itself provides the great opportunity to interpret the model predictions. In the introduction section authors made a statement that attention layers may limit the flexibility of model architecture when designing new models. Could they better explain this limit? Because recent state of the art models successfully work with long biological sequences and show better results then any other models (one example could be found here: <https://openreview.net/pdf?id=YWtLZvLmud7>). Authors should cover these limits more, that also related to the motivation of the LSTM choice.

We added a paragraph to our introduction to expand on attention and its limitations:

*“Attention mechanisms have been successful in recapitulating experimentally defined binding motifs, but require that the model be constructed with attention layers. This may limit the flexibility of model architecture when designing new models. For example, attention mechanisms are specific to neural networks. Simpler models, such as random forests and XGboost, may also be more suitable for some applications, and these cannot utilize attention. Also, while attention mechanisms are currently very effective, there is always a possibility that new architectures will emerge that make interpretations using attention infeasible. Beyond this, attention is a metric of the model itself, while SHAP values are calculated on a per input basis. By looking at the model through the lens of the inputs, we can understand the model’s “reasoning” behind any peptide. Attention mechanisms also do not enable dissection of interpositional dependencies between amino acids. Thus, new methods for model agnostic interpretation are desirable.”*

3. Another statement was made about the PoSHAP - adaptation of the SHAP method. It is hard to follow through the explanation of this adaptation - it is not clear what exactly is this adaptation. For example, Kernel SHAP from the original paper computes feature importance, in this paper authors compute the impact of each position, that is basically also the feature importances. Thus authors should better explain the statement about PoSHAP novelty. Will it be possible to use PoSHAP for any other model trained for the same purpose? If yes, for better reproducibility, authors should provide the place where exactly in the repo is the code for this. Also mathematical notations are missing in the Positional SHAP (PoSHAP) section - it is better to explain the adaptation with them to increase the understanding of the section.

We apologize for the ambiguous wording in the abstract stating that “PoSHAP adapts SHAP”. We have reworded this statement to “PoSHAP utilizes SHAP”. The novelty of this approach is taking the feature importance values calculated by SHAP and structuring them to include each position’s index to allow for the interpretation of biological sequences. As we demonstrate here, this allows for novel interpretations of previously published data and will enable model interpretation in future studies that learn from biological sequences. Although this is practically very simple, we are not yet aware of any examples in the literature that do this.

The following two SHAP force plots demonstrate the difference between using SHAP as-is versus PoSHAP. There is a demonstrated need for such a framework, considering the dearth of biological sequence model interpretation using SHAP and the ambiguity within biological sequence SHAP interpretation. For example, Meier *et al.*, Nature Communications, 2021 performed an analysis like our Figure S7C, which just shows the range of SHAP values per residue. Although we can learn something about which AAs are important based on the range of their SHAP values, SHAP as-is doesn’t reveal a motif. While our position indexing is a simple change, it enables all the rich, sequence dependent analysis we performed in this paper. We added the following text to the results section with this new supplementary figure:

*“PoSHAP utilizes the standard SHAP package but adapts the analysis by simply appending an index to each input and maintaining positional information after the kernelExplainer interpretation, which enables tracking of each input postion’s contribution to an output prediction (supplementary figure 5, showing force plot with and without index).”*

**Supplemental Figure 5. SHAP Forceplots Demonstrating PoSHAP Indexing.** Two forceplots were created with the SHAP forceplot method of the third peptide in the CCS testing set. (A) shows the plot with encoded inputs mapped to their amino acid. (B) shows the plot with the encoded inputs mapped to their amino acid and position. The addition of positional indexing removes the ambiguity of contributions, for example, glutamine having both a positive and a negative SHAP contribution to the prediction of the third peptide.

We have updated the repository to include a tutorial that demonstrates PoSHAP on provided data and explains how to use PoSHAP with your own model and data.

4. In the experimental section, authors first compare the results with previously known. For example, for the human MHC allele A*11:01 model PoSHAP analysis shows the similar results as was shown with another approach. Based on the provided explanation, it is not clear why PoSHAP is better than the previously published method. The advantage of the PoSHAP should be better explained.

We agree with the reviewer that the benefits of our approach should be as clear as possible. The referenced section of the paper is to validate our approach compared to another model interpretation technique. We added a new third paragraph to the discussion section to clearly explain the benefits of PoSHAP:

*“There are several benefits of PoSHAP over competing methods. First, PoSHAP determines important residues despite biases in the frequencies of amino acids (****Figure 5, Supplementary Figures 9 and 10****). PoSHAP is also applicable to any model trained from sequential data (****Figure 8****), and enables dissection of interpositional dependencies (****Figures 6 and 7****). Finally, we include a clearly explained jupyter notebook on Github that will take any model and dataset and perform PoSHAP analysis.”*

5. In the experimental section, after the PoSHAP performance verification, hypothesis generation was introduced. However, it is not clear how many hypotheses were generated; how many of them were known before; what kind of other categories are inside these hypotheses (unknown, possible and potentially interesting, etc).

*We are unsure as to how to quantify the number of hypotheses generated by our approach. In a sense, the SHAP value of each amino acid at each position within a heatmap represents a hypothesis of the contribution of that amino acid to the metric being predicted. Each significant interaction listed in the first three supplemental tables represents a hypothesis of the interactions between two given amino acids at two positions. To make these into testable hypotheses requires some analysis, as we have discussed. i.e. the two binding motifs (L-T-P, F-S-P) of A001, or the distance-type interactions within the CCS.*

6. The README section in the GitHub repo is not easily understandable. An additional explanation for each step is required (e.g., links to the folders where the calculated SHAP values, the trained models, all splits and all-important benchmarks are).

We have updated the README and repository to explain how to use PoSHAP, and explanations of each item in the repository.

**Minor comments**

1. The prior studies should be covered better (see Major comments).

We apologize for not better covering prior studies. We have significantly expanded the introduction by adding two new paragraphs and at least 10 additional citations.

2. The work consists of some typos, for example: "However, because many reports forgo model interpretation" - "t" is missed.

We did intend to use the word “forgo” not “forget” in that sentence. We have checked again thoroughly for spelling and grammar mistakes.

3. The hyperparameters table, hyperparameter search section should be moved to the supplemental material, that's technical details.

We moved this table to the supplementary materials.

Reviewer #3 (Significance (Required)):

Interpretation of the model results is an important topic for biology. New findings here could lead to new interactions opening, new drugs development etc. That is relevant for the applied ML Researches and computational biologists. This paper aims to provide a way to do it.
Because my field of interest and expertise lies in Machine Learning for healthcare, language modelling of biological sequences and Natural Language Processing, this work is of great interest to me. So I mostly evaluated ML methodology presented in the paper.
