# Supplementary material for: Positional SHAP (PoSHAP) for Interpretation of machine learning models trained from biological sequences
Source: PLoS Comput Biol. 2022 Jan 28;18(1):e1009736. doi: 10.1371/journal.pcbi.1009736 (PMC8797255; doi:10.1371/journal.pcbi.1009736)
Supplement: Attachment — Submitted filename: PoSHAP_review_commons-revision-coverletter-n2.docx [file pcbi.1009736.s020.docx]

September 2021

Dear Editorial Team:

I am writing to submit our revised manuscript for consideration in *PLoS Computational Biology* as an article, which is titled “Positional SHAP (PoSHAP) for Interpretation of Machine Learning Models Trained from Biological Sequences”.

On the following page please find our response to the one additional reviewer comment.

We thank you for your editorial guidance during the evaluation of this manuscript and look forward to hearing from you.

Sincerely,

_________________

Jesse G. Meyer, Ph.D.

Assistant Professor

Medical College of Wisconsin

Reviewer #1: Mathematical notations are still missing in Positional SHAP in explanation

We apologize for the confusion. There is no additional math required for positional SHAP analysis. The difference is in adding the index to each of the inputs, which allows us to track the positions of inputs in the subsequence SHAP analysis.We updated supplemental figure 5 to add the input sequence before and after adding the index to make this more clear:

**Supplemental Figure 5. SHAP Forceplots Demonstrating PoSHAP Indexing.** Two forceplots were created with the SHAP forceplot method of the third peptide in the CCS testing set. (A) shows the input and standard SHAP force plot with encoded inputs mapped to their amino acid. (B) shows the input with indexes and the PoSHAP force plot with the encoded inputs mapped to their amino acid and position. The addition of positional indexing removes the ambiguity of contributions, for example, two different glutamine residues and two different leucine residues each with both a positive and a negative SHAP contribution to the prediction of the third peptide.

Figure Files:

While revising your submission, please upload your figure files to the Preflight Analysis and Conversion Engine (PACE) digital diagnostic tool, https://pacev2.apexcovantage.com. PACE helps ensure that figures meet PLOS requirements. To use PACE, you must first register as a user. Then, login and navigate to the UPLOAD tab, where you will find detailed instructions on how to use the tool. If you encounter any issues or have any questions when using PACE, please email us at figures@plos.org.

We have uploaded the figures and included the output of PACE as an attachment in the submission.

Data Requirements:

Please note that, as a condition of publication, PLOS' data policy requires that you make available all data used to draw the conclusions outlined in your manuscript. Data must be deposited in an appropriate repository, included within the body of the manuscript, or uploaded as supporting information. This includes all numerical values that were used to generate graphs, histograms etc.. For an example in PLOS Biology see here: http://www.plosbiology.org/article/info%3Adoi%2F10.1371%2Fjournal.pbio.1001908#s5.

We uploaded all the data to zenodo and added the following statement to our methods “data and code availability” section:

The data including all points used to create the main figures are available from zenodo <https://zenodo.org/record/5711162#.YZaK-57MJ6I>

Reproducibility:

To enhance the reproducibility of your results, we recommend that you deposit your laboratory protocols in protocols.io, where a protocol can be assigned its own identifier (DOI) such that it can be cited independently in the future. Additionally, PLOS ONE offers an option to publish peer-reviewed clinical study protocols. Read more information on sharing protocols at https://plos.org/protocols?utm_medium=editorial-email&utm_source=authorletters&utm_campaign=protocols

There are no relevant protocols.

References:

Review your reference list to ensure that it is complete and correct. If you have cited papers that have been retracted, please include the rationale for doing so in the manuscript text, or remove these references and replace them with relevant current references. Any changes to the reference list should be mentioned in the rebuttal letter that accompanies your revised manuscript.

No changes were made to the references and we have checked the references list for correctness.
